# Lodgment of False Teeth in the Œsophagus

**Published:** 1864-01

**Authors:** John Swinburne


Lodgment of False Teeth in the Œsophagus.—*By John Swinburne*, M.D.—A few evenings since, Dr. Baily, of this city (Albany), called on me late at night, saying that one of his patients had swallowed two false teeth, with the gold plate attached, and that the mass was lodged in the Œsophagns between the os hyoid and the sternum.

From a case which had been previously brought to my knowledge, and which resulted fatally, I was naturally led to ruminate upon the most feasible and safe method of extraction. The result of the fatal case, a short history of which I will give by way of illustration, admonished me, that the bucket or sponge of the probang were inadmissible on account of the nature of the foreign body.

Then the merit of the gullet foreeps was discussed with favor, *i. e.* if the substance sought was within its reach or above the sternum.

Excision was in turn mentally discussed, and to such an extent that during our walk to the house, my friend thought me a very silent companion.

The patient was sitting up, and expectorating a large amount of thick, tough mucus, as the result of the irritation, and seemed very anxious as to the result. She was soon quieted. Upon examinatien, I found the foreign body occupying the position indicated by the doctor.

The gullet forceps were passed so as to find the metallic body, when the blades were openod so as to grasp the object. The first and second attempts at extraction failed, while the third effort was crowned with full success, and though the forceps lost their hold upon the arm of the gold plate, just as it emerged from the fauces, I had the satisfaction of seeing the body forcibly thrown forward into the mouth, greatly to the joy of our patient.

The plate was about one and a half inches in length and three-fourths of an inch in width, one clasp of which had been broken off, and which was the cause of the disaster. These were two front teeth, attached close together, while the two arms reach out some distance on either side, to obtain firm hold of the adjoining teeth, making the plate really much longer than it would otherwise have been.

The other case occurred in April, 1861.

In the course of some altercations, P. B., a young, strong and vigorous man, received a blow in the mouth, which broke the clasps of a gold plate containing four false teeth. This slid into the fauces, and produced much choking and strangling; soon this ceased in a measure; still he felt that it had not passed down beyond the sternum, though he stated to me that he swallowed fluid freely. The injury occurred about one, P. M., and he partook freely of sepon and milk at seven, P. M. Up to the time when the propang was passed, the pain felt was near the sternum or below the larynx. At eight o'clock, P. M., he proceeded to the office of a physician, when the button of the propang was passed and the patient forcibly withdrew the instrument. Four conseeutive attempts were made with the bucket, and twice did the patient forcibly withdraw the instrument. The attendant pain he described as being very severe, followed by vomiting, and spitting of much blood at the time. This continued freely all night, and most of next day. Much clotted blood was vomited. At eight and a half o'clock the same evening, the patient called upon another physician, who simply explored the parts with the sponge probang, discovered the nature of injury, and desisted from any further effort. About eleven, P. M., emphysema was discovered, over a space equal to about ten inches in diameter, and extending over the upper end of the sternum, face and neck, and most upon the right side, I saw the patient the next day, and found the condition as described. The fact that the lung had been wounded, either from the buckct of the propang or the gold plate, coupled with other symptoms such as dyspnœa, etc., induced me to advise non-interference. Tne subsequent post-mortem confirmed the prudence of the advice.

This patient died some days subsequent, after severe suffering, being unable to swallow solid food, and fluids only in a limited quantity, while the dyspnœa remained exceedingly oppressive.

*Dissection.*—Post-mortem about twenty-four hours after death. The emphysema still existed to some extent. There was much swelling of the neck, which, upon dissection, proved to be composed principally of imperfectly formed pus, holding in suspension a large amount of flocculent, fibrous tissue, looking not unlike shreds of linen or cotton fiber, mixed intimately with pus; the abscess extended several inches in front, from the sternum to the os hyoid, isolatimg the œsophagus for about two inches, and burrowing among the muscles of the right side of the neck. The plate was found obliquely across the neck, just behind the superior border of the sternum, its concavity looking forward and upward, its right extremity was the lowest, being in contact with the lung.

The œsophagus was ulcerated through, on both sides, so that all the fluid food taken passed out of its right side, and thence run into and filled the pleural cavity. Of course, pleuro-pneumonia supervened, and resulted in compression and destruction of the lung proper.

I found the plate, as before stated, contained four teeth, three together on the left side—a space of two teeth intervening, and the fourth tooth was placed on a prong or shaft —both clasps were broken, leaving the remaining plate about one and a half inches in length, and about three-fourths of an inch wide, while the edges of the plate were so sharp as readily to cut through the tissues, upon the application of any considerable force, sufficient to change the longitudinal axis to a transverse one, as would be the case if the bucket of the probang were to be caught on the inferior border of the plate near its center.

The question naturally arises, would the same result have followed if the gullet forceps had been used? Would the blades of the forceps occupy the same amount of space as the bucket? Would not the long axis of the plate naturally correspond with that of the œsophagus? and would not the passage of the bucket by the side of the plate naturally force it out of the longitudinal axis? Could the bucket be made to pass by so large a body without rupturing the œsophagus? Is there any obstable to the passage of this or any other body at this point, any more than there is at any point above? If not, then why was not the œsophagus cut above the point indicated? That the wound in the œsophagus was not the result of ulceration alone, is evidenced by the fact that much blood and vomiting followed the efforts at extraction, as did also emphysema, showing that the wound in the lung and œsophagus was produced nearly simultaneously, and was probably the result of either the spasmodic vomiting or the efforts at extraction. In a case of this kind the forceps present this advantage, that they need not be passed by the foreign body, but rather grasp the superior presenting part, and thence keep the long axis of the plate parallel. By this means, it is possible that the material injury to the soft parts might have been avoided. By this I do not mean to cast any imputation, since any of us, without due reflection, would unquestionably fall into the same error, if error it was.

I frankly confess, that without the experience arising from this case, I should have applied the bucket to the case herein reported as having been so successfully relieved by the gullet forceps. The probabilities are, that no treatment would have changed the status of the fatal case; still it is equally important that we should bear in mind the dangers which beset the extraction of this kind of foreign substance; and, also, the importance of using the kind of instrument which is the most effectual and the least dangerous to life.

I have the verbal history of two cases which proved fatal from suffocation before any relief could be extended, and all from a little neglect in wearing plates with but one clasp. Before any attempt at removal be tried, the nature of the foreign body should be taken into consideration. If it be of such a nature as to be readily passed through the alimentary canal, and no objections to its passage either down the œsophagus or through the alimentary canal, then it should be passed down at once.

On tne contrary, if it be a large or irregular mass, and of such a nature that it can not be safely passed through the œsophagus, nor pass readily out of the stomach, then it should be extracted, and that too by some instrument like forceps, which can seize the superior portion, and thus be extracted as it prsses down, *i. e.*, the long axis of the foreign body shall correspond to the long axis of the œsophagus.

[*Med. and Surg. Reporter.*

